# The General and the Local in Dental Pathology

**Published:** 1893-04

**Authors:** J. Smith Dodge

**Affiliations:** New York, N. Y.


### The General and the Local in Dental Pathology.

BY J. SMITH DODGE, JR , M.D., D.D.S , OF NEW YORK, N. Y.

Read in the Section of Oral and Dental Surgery, at the Forty-third Annual Meeting of the American Medical Association, held at Detroit, Mich., June, 1892.

The whole field of pathology is divisible into the general and the local; and the opinions of pathologists have fluctuated many times in assigning the controlling power to one or the other.

Ignorant minds necessarily look at the seat of pain or other disturbance as containing the disease. It was considered a great scientific advance when disease was pronounced an affection of "the humors," meaning all the liquids of the body, and therefore conceived as general with local manifestations. In our time the cellular pathology of Virchow has given a strong impulse again toward the more local conception, and bacteriology seemed for a while about to convert all pathology into the local action of microbes. But the opinion grows that it is not so much the bacteria as their ptomaines, diffused by the hæmatic and lymphatic circulations, which cause disease; and this may bring us back again to the humoral theories, with the great advantage that we have discovered the *materies morbi*.

Dental pathology is so recent that it can hardly be said to have shared all these fluctuations; but it has room enough for them all, and its short history shows now the general and now the local view predominant.

The earliest modern dentists were first of all physicians, several of them being among the great lights of medicine, and they naturally viewed all affections of the teeth, not of mechanical origin, as symptomatic of general disorder. But when dental operations had been invented the processes were so obviously local both in causes and results, that they fell speedily into the hands of men who were merely skilled mechanics, and the general relations of the art were almost neglected or treated in the most empirical manner.

A great change resulted from the labors of Dr. Harris and the founding of the Baltimore College of Dentistry, which was the birth of dental education. For many years the constant effort of educated dentists was to remove the stigma of a mechanical art and to assert their affiliation with medical men. This effort naturally brought forward again whatever could be alleged as connecting the teeth with the general system, whether as exerting or suffering pathological influences. And there was a time, midway in this struggle, when cautious men were inclined to think that the manual skill and the practical usefulness of a dentist were pretty certainly in inverse ratio to his scientific knowledge of medicine. Indeed, a contrast used to be drawn between European and American dentists along this line; the Europeans being confessed the more scientific, but the Americans far more practical.

Such work as Harris', however, could not fail, and by slow degrees dentistry has come to its own. We no longer need to ransack the records of medicine for rare and doubtful cases which may show the reciprocal relation of teeth to nerve-centres, in order to prove that we deal with vital organs intimately associated with the general welfare of the system. Every division of the dental field is now known to demonstrate this abundantly; and a suggestive outline of the familiar facts is all we shall need here.

It is not worth while to dwell on mere curiosities. Almost every organ of the body has at some time been reported as having pathological relations with the teeth; but it may be doubted whether all these cases could have stood the test of modern research. At any rate the very rare ones can never be anticipated and seldom recognized, and may well be left out of our view.

Neither need we take up the sequels of inflammatory action produced by diseased teeth in the adjacent tissues; the periosteal tumor which sometimes long precedes the breaking of a chronic alveolar abscess; burrowing fistulæ which occasionally open far from the tooth; alveolar necrosis; affections of the antrum, etc. There is no mystery about these results and there can be no advantage in going over them here.

The remote relations of the teeth which still need study are effected either by the nerves, (whether the teeth are on the causative or the resultant side), or by general cachexias not well understood.

There can be no doubt that the constitution and the structure of the teeth partake of the general conditions of the system during the formative stage. This relation is a little obscured by the fact that the internal condition of teeth does not so readily fluctuate with changes of the general health as do most other tissues; because the process of nutrition and interstitial change is exceedingly slow. But during the formation of the teeth they are as easily influenced as other organs, and their subsequent condition, especially the difference often found in the same mouth between teeth of different periods, may frequently be accounted for by inquiring into the health of the child at the time when each order of teeth was forming.

A relation so easily established authorizes us to believe that a similar cause may some day be made out for more mysterious deficiencies of dental constitution, and pathological conditions be thus explained from within, for which no study of their surroundings shows any cause.

But the history of teeth is not wholly determined by the circumstances of their formation. Many observations have proved that a slow circulation pervades the dentine, maintaining a correspondingly slow process of renewal and interstitial growth. This process certainly produces at times increased density, and the presumption almost amounts to certainty that it sometimes retrogrades and causes deterioration. The evidence of this is not easy to disentangle, but it grows in distinctness. It has long been observed that the teeth rapidly decay during or immediately after some attacks of prostrating sickness and some pregnancies. The cause usually assigned has been a pathological acidity of the buccal fluids; but it is now established by researches in bacteriology, that except at the very beginning caries depends very little on external conditions; and therefore, if the long recognized effects of sickness and pregnancy are facts, some cause must exist which acts within the tooth. And such a cause does exist which is exactly in line with our present study. Experiments made by the writer some years ago, but never published, showed that when decay occurs in fairly well formed teeth, a zone of dentine immediately surrounding the cavity of decay has a higher specific gravity and yields more ash than a similar section from a sound side of the same tooth. This necessarily means a new deposit of lime salts apparently resulting from the irritation of decay. Now such a deposit in itself is of course a local affair, but it implies a heightening of vital activity which must be traced back to nerve centres. And it may well be assumed that besides the increase of density, the increased vitality of the dentine hinders, *pro tanto*, the ravages of the bacteria. If then this be the normal check upon decay, anything which so cripples the nerve centres as to prevent this opposition, leaves the tooth defenceless to its enemies, and destruction is far more rapid. Now to this failure of normal inhibition rather than to any local cause, the quick decay accompanying disease and pregnancy must be ascribed.

Another field of quick decay reinforces this conclusion. In advanced life, when the general strength is impaired, teeth which have borne the strain of a lifetime are seen to give way suddenly to decay, so that in a few months there will be little left of the crown except the enamel, the dentine being decalcified in mass. Of course something must have happened to make this possible, and not only general reasoning but the fact that these teeth are remarkably insensible under the excavator, indicates that the nerves which supply them have almost ceased to work.

From all these considerations it seems fair to conclude that the decay of teeth is far from being a purely local matter, and is greatly determined by the nerve-centres from which the teeth are supplied.

Another branch of dental pathology which was long treated as merely local, has come to appear still more dependent on general conditions—the whole group of pericemental affections loosely classed as Rigg's disease. Once every condition, from a slight marginal congestion to the suppurative inflammation of the whole membrane was called the result of "tartar;" and under this name were included both salivary calculus and the scales and stars of pericemental exudate which occur at all parts of an affected root. With this view of the cause it could not be doubted that some kind of local treatment would cure the disease; and the remedies warranted never to fail have been countless.

The fact is, however, that extreme deposits of salivary calculus, even masses as large as the tooth, are frequently found to have caused no worse trouble than a superficial inflammation with some absorption of the gum; while deep-seated lesions of the most fatal kind occur in mouths which have been kept carefully clean. There is some cause further back. Of course bacteria are invoked to explain it all, and they doubtless play a large aggravating part. But they cannot explain why one mouth welcomes and another rejects them under the same visible conditions.

This writing has been interrupted to see a case which perfectly illustrates. Two molars have been driven apart by the blow of a wedge-shaped antagonist. At every meal food packs tightly against the intervening gum, till it has receded far beyond the original level of the alveolus, forming a deep pocket between the teeth. Could any conditions be more inviting to bacteria—the deep, protected nidus, the ever renewed supply of food never quite removed, the air and heat and moisture of the mouth? And yet neither tooth shows the least sign of pericemental disease, and the depressed surface of the interdental gum is pale and healthy as the hard palate. Such cases, not very infrequent, prove that the whole is not stated when we have spoken of bacteria. The opinion of dentists is therefore inclining towards one or another constitutional state as the predisposing and sustaining cause of pericemental trouble. We do not yet know much about it. Perhaps gout or rheumatism is at the bottom of it; perhaps the condition, not well understood, which promotes nasal catarrh.

To these two conspicuous topics might be added odontalgia; but of this so much is superficially known and so little profoundly, that it is hardly profitable for discussion. It is enough to say that the pains of the teeth are as frequently unaccountable as any others. The great majority, it is true, are due to local irritation traceable if sufficient diligence is used. But the commonest perplexity of dental practice is that one may do so much in one mouth without painful sequels, while so small a portion of similar treatment in the next mouth will prove disastrous.

Nor has as much been anywhere said as might be of fugitive and unaccountable pains in the teeth. It often happens that the most searching examination discovers no local cause and that nothing in the subsequent history throws any light on the matter. Of course such phenomena are to be referred to reflex-action. The fifth pair present so many points of possible irritation, and this is known to pass so readily from one branch to another, that it would be strange indeed if the teeth did not often complain of disturbances quite remote from them.

Irregularities of position constitute another topic which might occupy us. When all the cases have been subtracted which can be explained by mechanical causes, a few remain which are partly ascribed to heredity and partly left without any explanation. It is probable that further study of these will bring to light constitutional causes, perhaps reaching back to fœtal life. But the whole matter waits for patient investigation.

This review, then, while it shows very much yet to be accounted for, also shows that dental pathology is but a part of general pathology and can by no means be studied alone.

And yet when we turn to the dentist's daily practice, we are surprised to find how much all this may be left out of account, how overwhelmingly the troubles which he treats admit of purely local explanation and cure. Indeed, the man who broods beside his operating chair over remote causes and turns his best attention to general remedies, will by-and-by find that chair empty, and will learn that he is a practical failure.

Let us next view the matter, therefore, from this side and see to how great a degree dental pathology is local.

Nobody doubts that uncleanliness of the mouth promotes decay; and no theory of electro-chemical action nor any discovery in bacteriology can shake this long established result of observation. It is equally certain that the careful excavation and filling of any given cavity always retards and frequently ends forever the morbid process.

It is worth while to add that when filling is unsuccessful and the question is raised whether the tooth or the operation is at fault, most practical dentists will incline, (if the operation is not their own), to suspect a fault in the filling.

One cannot speak quite so forcibly of local causes in pericemental disease. And yet the results of local treatment are so often beneficial that local causes must play a large part, and the attempt to trace these disorders back wholly to constitutional causes must not only fail but must seriously interfere with the discovery of improved local treatment.

As for the local causes of odontalgia, they are very numerous and sometimes so hidden that a diagnosis of remote causation is always to be looked upon with suspicion and most rigorously questioned. And this, with a little less emphasis, is to be said of dental irregularities.

It might seem that such a survey as has been here made leaves the whole subject in confusion, and dentists must again divide into two opposing camps as in earlier days—the medical dentists and the operators.

But this can never be. There is no room for such strife, and if it could be attempted, the contest would be too unequal and would be speedily fought out. For all theories are subject to the test of facts; and the fact is that the reason of our existence as dentists, the warrant for the high estimation in which our art has finally come to stand, is the possibility of relieving dental troubles by local operations. No amount of diagnostic acumen or of scientific learning will make a man a successful dentist who cannot give prompt comfort and safe assurance of future usefulness to the mouths of his patients.

The writer hopes to be particularly understood as building, whether at the chair or in an essay, on the intelligent, patient, and skillful performance of dental operations, as the corner-stone of dentistry. Whatever we may discover or invent, the day will not come in our time when there will not be ravages of past disease to repair and conditions of present disorder to cure.

It will be indeed a happy day for the science of the teeth if there ever arise men with the leisure and the learning necessary to work out the meaning of innumerable facts separately collected and recorded by practicing dentists. A single such man devoting his life to this study and to the verification of such conclusions as might be drawn, would make for himself a name and for dental science new means of usefulness, so great that we can as yet hardly estimate them. But no man can possibly do this who is tied to the operating chair; and the zeal that works without fee combined with the fortune which makes this possible, does not yet seem to have fixed its attention on dental science.

Meanwhile what can we, who must do our daily tasks, also do towards broadening the horizon of dentistry, towards hastening the happy day when dental disorder shall be rather prevented than cured?

Dentists have long nursed this hope, and the present paper aims to add another impulse in that direction.

It is no violent exercise of imagination to fancy the dentist of the future receiving a new patient. The chair, the mouth-glass, the probes will all be there, and the examination, tooth by tooth, as minute as now. Then will follow a series of questions covering the ancestry, the habitual surroundings, the personal history and habits of the patient, and at last the dentist will give his judgment. "You have such and such defects of the teeth," he will say, "which will all be duly corrected. You have this or that constitutional weakness which has caused or promoted your dental troubles. Or your diet, your occupation, your habits are at fault, and it will be necessary to make changes thus and thus." After six months perhaps the patient will return, the examination will be repeated, the regimen approved or changed as results may have appeared. In a few months or years the patient will have ceased to have any further dental disasters beyond the inevitable wear and tear of hard used organs; and after two or three generations a race will be produced in whom dental disorders will rank with pulmonary or ocular diseases, always possible but commonly escaped.

No thoughtful dentist will say the picture is too flattering, yet how delightful it is! And how are we to realize it? By holding fast the conviction that behind the local cause and effect there are powerful general causes; that these are not simple and manifest but difficult to trace and much interwoven; and that therefore they are not to be discovered by catching up some theory of phosphates, or rheumatism, or bacteria, but by the most patient accumulation and comparison of cases which now and then give some valuable hint, and a slow induction of broad facts and general principles. As it will take several generations of patients properly guided to produce the dentition of the future, so it will take several generations of educated, diligent dentists to acquire the wisdom which may guide.

Perhaps it will seem a scanty outcome of so many words, but the conclusion of the whole matter is this: Besides the patient work of local treatment and repair from which we cannot escape, we must vigilantly watch and search for remote causes, we must question and compare unweariedly, we must nurse a wholsome suspicion of theories which explain too easily or too much; and we must count it a life well spent if, besides innumerable daily ministrations of comfort to our patients, we can at last contribute to our profession one step forward in a knowledge of the fundamental causes of dental pathology.

### DISCUSSION.

Dr. Talbot agreed with the essayist that the diseases of the teeth were often intimately connected with diseases of the system, and instanced two cases of patients suffering with pyorrhœa alveolaris who had been under his care, but for whom he had not found it possible to effect any permanent good. The disease of the teeth affects the general system, and the disease of the system affects the teeth. In both cases he had advised long ago that the teeth should all be extracted and artificial dentures inserted in their place. All the teeth are sound, and the patients would not consent to lose them, though it is the only course by which they can expect to enjoy life. The patients have now become so physically run down that it is scarcely possible for them to get from their homes to their offices. They were of gouty diathesis, and when they had excess of acid in the urine the teeth would be surrounded with pus, and so loose that they scarcely could be kept in their sockets. The age of the patients was about thirty-five to forty-two years, and the trouble had been running on for fifteen or more years.

To understand such cases it is necessary to study the whole systemic condition. So also in neuralgia we often find the cause in a distant part of the body; for instance, it may be in the uterus, it may be from dyspepsia or a gouty or rheumatic condition.

Dr. Marshall said that one of the patients of whom Dr. Talbot spoke had come into his hands. He first looked for constitutional cause for the trouble. There was no tartar on the teeth. He made up his mind the patient was gouty, and upon examining the urine found a large excess of uric acid. Upon inquiring into his habits it was learned that he was an excessive meat-eater. Being advised to limit himself in this regard, he improved, but he will not give up his meat to the extent necessary to avoid the trouble, so now and then he returns for local treatment, which is given, and he is told to return the next day, but he never does. When he has the severe suppuration there is always excess of uric acid; no doubt the excess of uric acid or gouty condition affects the suppuration as a cause.

